# Bioabsorbable, subcutaneous naltrexone implants mitigate fentanyl‐induced respiratory depression at 3 months—A pilot study in male canines

**DOI:** 10.14814/phy2.16176

**Published:** 2024-08-08

**Authors:** Robert L. Joyner, Joseph A. Hollenbaugh, Donald D'Aquila, Marc Fishman, Steven M. Cohen, Veera Holdai, Jeffrey D. Benner

**Affiliations:** ^1^ Richard A. Henson Research Institute, TidalHealth Peninsula Regional Salisbury Salisbury Maryland USA; ^2^ Labcorp Greenfield Indiana USA; ^3^ The Department of Psychiatry Johns Hopkins School of Medicine and Maryland Treatment Centers Baltimore Maryland USA; ^4^ The Drug Delivery Company LLC Dba Akyso Salisbury Maryland USA; ^5^ The Department of Mathematical Sciences Salisbury University Salisbury Maryland USA

**Keywords:** bioabsorbable, canines, fentanyl, implants, naltrexone, respiratory depression

## Abstract

The aim of this study is to determine if extended‐release, bioabsorbable, subcutaneous naltrexone (NTX) implants can mitigate respiratory depression after an intravenous injection (IV) of fentanyl. Six different BIOabsorbable Polymeric Implant Naltrexone (BIOPIN) formulations, comprising combinations of Poly‐d,l‐Lactic Acid (PDLLA) and/or Polycaprolactone (PCL‐1 or PCL‐2), were used to create subcutaneous implants. Both placebo and naltrexone implants were implanted subcutaneously in male dogs. The active naltrexone implants consisted of two doses, 644 mg and 1288 mg. A challenge with IV fentanyl was performed in 33 male dogs at 97–100 days after implantation. Following the administration of a 30 μg/kg intravenous fentanyl dose, the placebo cohort manifested a swift and profound respiratory depression with a ~50% reduction in their pre‐dose respiratory rate (RR). The BIOPIN NTX‐implanted dogs were exposed to escalating doses of intravenous fentanyl (30 μg/kg, 60 μg/kg, 90 μg/kg, and 120 μg/kg). In contrast, the dogs implanted with the BIOPIN naltrexone implants tolerated doses up to 60 μg/kg without significant respiratory depression (<50%) but had severe respiratory depression with fentanyl doses of 90 μg/kg and especially at 120 μg/kg. Bioabsorbable, extended‐release BIOPIN naltrexone implants are effective in mitigating fentanyl‐induced respiratory depression in male canines at about 3 months after implantation. This technology may also have potential for mitigating fentanyl‐induced respiratory depression in humans.

## Graphical abstract

The study aims to evaluate if extended‐release, bioabsorbable subcutaneous naltrexone (NTX) implants can mitigate respiratory depression following intravenous fentanyl administration. Placebo group dogs experienced a ~50% reduction in respiratory rate after a 30 μg/kg fentanyl dose, while BIOPIN NTX‐implanted dogs tolerated doses up to 60 μg/kg without significant respiratory depression. This suggests BIOPIN NTX implants effectively mitigate fentanyl‐induced respiratory depression in male canines, with potential applicability in humans.

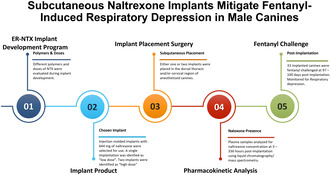

## 1 INTRODUCTION

The opioid crisis in the United States has led to a significant increase in fatalities, as nearly 107,000 persons in the U.S. died from drug‐involved overdose in 2021, including illicit drugs and prescription opioids (Edinoff et al., 2021; National Institute on Drug Abuse, 2023). Illicitly produced fentanyl, which is 50–100 times more potent than morphine and less responsive to emergency overdose reversal with naloxone than heroin, contributes to this surge (National Institute on Drug Abuse, 2021; Schiller et al., 2024; Wilson et al., 2020). For patients with opioid use disorder (OUD), withdrawal from opioids is aversive and often leads to relapse with resumption of opioid usage. Relapse may also occur when memories or cues associated with previous opioid use trigger cravings for the drug (Heinsbroek et al., 2021). Naltrexone, a competitive antagonist of mu opioid receptors, offers an effective pharmacological solution by reducing cravings and preventing fatal respiratory depression. But adherence remains a challenge with the currently available oral or monthly injectable formulations (Edinoff et al., 2021; Sudakin, 2016).

Extended‐release naltrexone (NTX) implants, offering longer durations of active medication delivery, show promise in addressing adherence issues (Colquhoun et al., 2005; Colquhoun, 2013; National Library of Medicine, 2019). Currently, the longest‐duration formulation of injectable naltrexone, Vivitrol (Alkermes), lasts only about 1 month (FDA, 2006). We are developing an extended‐release, bioabsorbable naltrexone implant that can be implanted in the clinic and will release therapeutic levels of NTX for 6–12 months. The continuous release of NTX from the implant will improve compliance for OUD patients by controlling opioid cravings and withdrawal symptoms, promoting retention in treatment, and may prevent fentanyl‐induced respiratory depression if a patient relapses and uses fentanyl.

It is crucial to determine if these formulations can effectively counter the potency of fentanyl analogs, which now dominate the illicit opioid supply and overdose cases. As part of a program to develop a better extended‐release naltrexone implant, we created and tested NTX formulations made with release‐controlling polymers that are safe and well‐tolerated in humans (DeStefano et al., 2020; Food and Drug Administration, 2021). We recently demonstrated that extended‐release, bioabsorbable, subcutaneous naltrexone (NTX) implants inhibit respiratory depression after an IV injection of fentanyl in rodents as compared to placebo implants, 3.5 months after implantation (Benner et al., 2023).

This study aimed to validate the efficacy of subcutaneous NTX implants in larger mammals, specifically canines, which is required for the toxicology studies that the FDA requires before allowing phase 1 clinical trials to proceed in humans. Therefore, we aimed to fortify the findings of our rodent study in a larger mammal before proceeding with human trials for OUD. (Benner et al., 2023). We wanted to know if extended‐release NTX implants, inserted months in advance, could effectively mitigate fentanyl‐induced respiratory depression following intravenous fentanyl in canines, as we have observed in rodents.

## 2 METHODS

### 2.1 Implant fabrication

**FIGURE 1:**
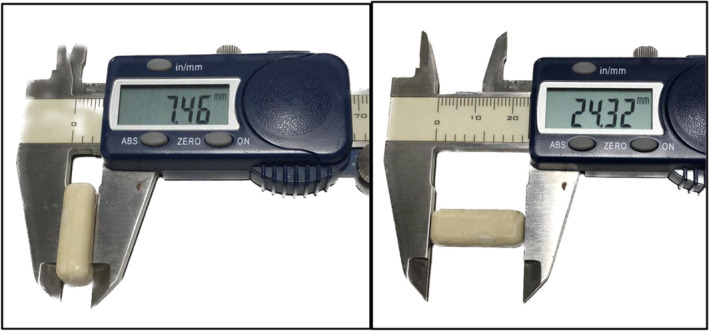
Photographs of representative BIOPIN Implant. A caliper shows the implant's dimensions in mm.

As a part of our extended‐release NTX implant development program, different release polymers and different doses of NTX were evaluated. The implants were fabricated with either PDLLA or PCL. Each implant was loaded with different doses of naltrexone, 0 mg (placebo) or 644 mg of naltrexone base (SpecGx LLC, Webster Groves, MO, USA). The implants were fabricated using the method described in USPTO # 11,197,819 B1 (Benner et al., 2021). The different implant formulations were identified as a BIOPIN, an abbreviation taken from the first letters of their descriptive name: BIOabsorbable Polymeric Implant Naltrexone. In brief, the naltrexone and the release‐controlling polymers were mixed and heated to 170–180 degrees centigrade. The molten mixture was injection molded into a rectangular mold to form implant measuring 0.75 cm × 0.98 cm × 2.43 cm with a volume of 1.79 cc and containing 644 mg of naltrexone (Table 1 and Figure 1). After cooling, each implant was removed and packaged for sterilization. The implants were then sterilized with ethylene oxide. For the experimental intervention, a single (644 mg) NTX device implantation was considered “low dose.” A “high dose” NTX intervention consisted of two 644 mg naltrexone implants (1288 mg of NTX) subcutaneously placed into the animal (total volume of 3.58 cc).

**TABLE 1:** Formulations of BIOPIN implants with naltrexone doses.

BIOPIN number	Release controlling polymer (ratio if blended)	Number of canines/dosage	Naltrexone dosage (mg)	Volume of BIOPIN implant(s) (cc)
1	PDLLA	4	0	3.58
		4	644	1.79
		6	1288	3.58
2	PCL‐1 (0.4 dL/g)	4	0	3.58
		4	644	1.79
		6	1288	3.58
3	PCL‐2 (0.8 dL/g)	4	0	3.58
		4	644	1.79
		6	1288	3.58
4	PCL‐1/ PCL‐2 (50:50)	4	0	3.58
		4	644	1.79
		6	1288	3.58
5	PDLLA/PCL‐1 (70:30)	4	0	3.58
		4	644	1.79
		6	1288	3.58
6	PDLLA/PCL‐2 (60:40)	4	0	3.58
		4	644	1.79
		6	1288	3.58

Abbreviations: cc, cubic centimeters; PDLLA, poly‐D‐L‐lactide; PCL, polycaprolactone.

### 2.2 Animals

As part of a larger study, 84 male beagles (naïve) were purchased for use in a study at LabCorp Greenfield site. Thirty‐three of these animals were allocated to the fentanyl challenge experiments. Animals were 6 months to 3 years old and were acclimated to the test facility for at least 14 days prior to initiation of dosing. All procedures followed applicable animal welfare regulations and were approved by LabCorp‐Greenfield Institutional Animal Care and Use Committee.

### 2.3 Implant placement surgery

Surgery was performed under aseptic conditions, and sterile surgical instruments were used. The animals were anesthetized with butorphanol IV and dexmedetomidine. A small incision was made in the skin at the cervical thoracic area, and a small subcutaneous pocket was fashioned caudally from the incision for the implant to be placed. The implants were placed in the dorsal thoracic and/or cervical regions (lateral to the spinous processes) of the animals, as far away from incision as possible, to minimize implant erosion and/or dehiscence. The skin was closed with skin sutures. After the surgical procedure, each animal was injected with atipamezole and monitored until fully recovered.

### 2.4 Pharmacokinetic analysis for naltrexone levels

Blood was collected into tubes containing potassium EDTA as the anticoagulant. Plasma was harvested and stored in a freezer, set to maintain −60 to −80°C. Plasma samples were analyzed for naltrexone from 3 to 336 h post‐implantation for BIOPIN‐1, 2, 3, 4, 5, 6, using a qualified liquid chromatography/mass spectrometry (LC‐MS/MS) method at the Labcorp‐Madison site.

### 2.5 Fentanyl challenge

Fentanyl citrate salt (Sigma‐Aldrich, St. Louis, MO) was diluted in 0.9% NaCl solution to a dose that ranged from 30 to 120 μg/kg per dose. The dose for each animal was calculated from the most recently recorded body weight. Fentanyl was administered by intravenous (bolus) injection via peripheral vein. Animals were segregated into different cohorts for fentanyl administration, as denoted in the results section. Naïve canines (no implants) were used to determine the *minimum inhibitory dose of fentanyl* that would cause a reduction in the baseline respiratory rate by >50% from the pre‐dose baseline. The start of each fentanyl challenges was staggered to allow monitoring and provide rescue treatment when needed.

### 2.6 Respirations

Respiratory rate was recorded for each animal prior to administration of fentanyl (pre‐dose) and at predose, 30 and 90 s. Respiration rate was recorded in breaths/minute. Panting was noted episodically in some animals (Tables 3 and 4), both during the pre‐dose and post‐dose periods, which prevented the counting of respiratory rates. Animal cohorts with different implants were challenged with fentanyl at 30, 60, 90, or 120 μg/kg per dose until respiratory rate was depressed to a point requiring rescue treatment with vigorous shaking or IV naloxone. The supervising veterinarian would not any additional challenge experiments at the 120 μg/kg dose due to concerns about the animal's safety.

### 2.7 Statistical analysis and graphing

The normal respiratory rate for dogs is 15–35 breaths per minute for an animal at rest. A respiratory rate of >40 breaths per minute is abnormal and is often described as “panting” (Lake Normal at Mooresville Animal Hospital, 2023). For this study, any respiratory rate >40 bpm obtained before (pre‐dose) or during the fentanyl challenge was recorded as “panting.” Since the panting measurements could not be assigned a numerical value, the panting data points were not included in the graphs or the statistical analysis. The panting values are shown in Tables 3 and 4.

Statistical analysis was performed with an ANOVA to test for significant differences in respiratory rate depending on the four doses of fentanyl tested (placebo, 1×, 2×, and 3×) and three‐time intervals (pre‐dose, 30 and 90 s) using a two‐factor ANOVA with four levels for each factor. It is important to note that the placebo group consisted of six animals, two naïve canines (no implants) and four placebo implanted canines with plasma NTX levels of 0 ng/mL, rendering all six animals pharmacologically equivalent for the purpose of the fentanyl challenge. The fifth dose of fentanyl (120 μg/kg) was not included in the analysis because this dose was only given to one animal. A *p* value of <0.05 was considered statistically significant. The primary end point was prevention of a >50 ± 2% reduction in the pre‐dose respiratory rate. Standard deviations (SD) are noted in tables. Data were graphed using Minitab statistical software (Minitab, LLC State College, PA, USA) and GraphPad Prism version 8 (GraphPad Software, San Diego, California, USA).

## 3 RESULTS

The study utilized male beagles with mean body weight of 9.5 (±2.0) kg and aged 0.5–3 years at the time of implantation. The researchers implanted all six different BIOPINs in the canines, comprising combinations of PDLLA and Polycaprolactone (PCL‐1 or PCL‐2), either alone as placebo or loaded with naltrexone as an active component (Figure 1 and Table 1). The naltrexone implants were tested in two doses: a “low” dose of 644 mg with a single implant and a “high” dose of 1288 mg with two implants inserted side‐by‐side in the same anatomic pocket.

### 3.1 Pharmacokinetics of the naltrexone implants

**FIGURE 2:**
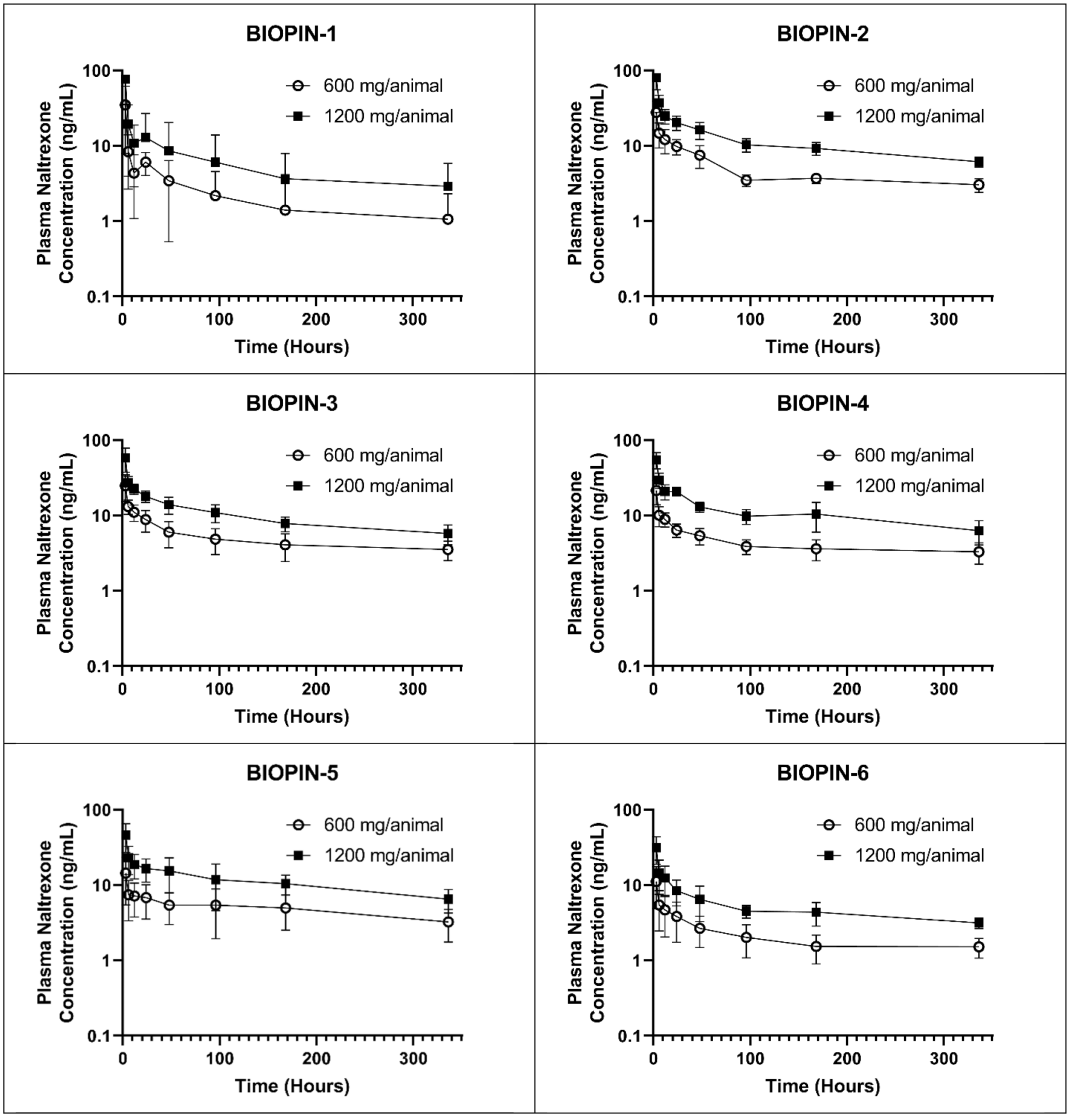
Acute pharmacokinetic evaluation of the six different BIOPIN implants. Plasma concentration of naltrexone (ng/mL) were evaluated from 3 through 336 h post‐implantation for BIOPIN‐1, 2, 3, 4, 5, and 6. Low‐dose (644 mg; open circle) and high‐dose (1288 mg; filled square) BIOPIN implants are displayed.

The pharmacokinetic profile of all six different BIOPIN NTX implants from 0 h through 336 h post‐implantation is displayed in Table 2 and Figure 2. The results showed that all six BIOPIN NTX implants initially exhibited a burst release, leading to high naltrexone plasma concentrations at 3 h post‐implantation. This burst was followed by a gradual decline, transitioning to a constant and steady release profile up to 336 h for all BIOPIN implants.

**TABLE 2:** Mean (SD) toxicokinetic results for plasma naltrexone concentrations following subcutaneous BIOPIN implantation.

BIOPIN type (dose of NTX/implant—mg)	*C*_max_ (SD) ng/mL	*T*_max_ (SD) h	AUC_0‐4292_ (SD) h*ng/mL (197 days)
BIOPIN‐1 (644 mg)	38.5 (21.3)	632 (1260)	11,500 (4300)
BIOPIN‐1 (1288 mg)	80.7 (31.7)	235 (567)	23,800 (9140)
BIOPIN‐2 (644 mg)	27.8 (11.5)	3 (0)	6940 (NA)
BIOPIN‐2 (1288 mg)	80.5 (25.2)	3 (0)	12,300 (3560)
BIOPIN‐3 (644 mg)	24.9 (9.69)	3 (0)	6110 (1630)
BIOPIN‐3 (1288 mg)	58.0 (20.4)	3 (0)	9710 (3900)
BIOPIN‐4 (644 mg)	21.5 (7.61)	3 (0)	6000 (1220)
BIOPIN‐4 (1288 mg)	54.9 (13.3)	3 (0)	16,000 (4130)
BIOPIN‐5 (644 mg)	19.2 (10.3)	2090 (2420)	16,200 (5920)
BIOPIN‐5 (1288 mg)	46.1 (19.0)	3 (0)	31,800 (13,100)
BIOPIN‐6 (644 mg)	12.1 (5.34)	1230 (1660)	13,400 (2340)
BIOPIN‐6 (1288 mg)	37.8 (16.8)	1450 (2290)	28,200 (8100)

Abbreviation: SD, standard deviation.

**FIGURE 3:**
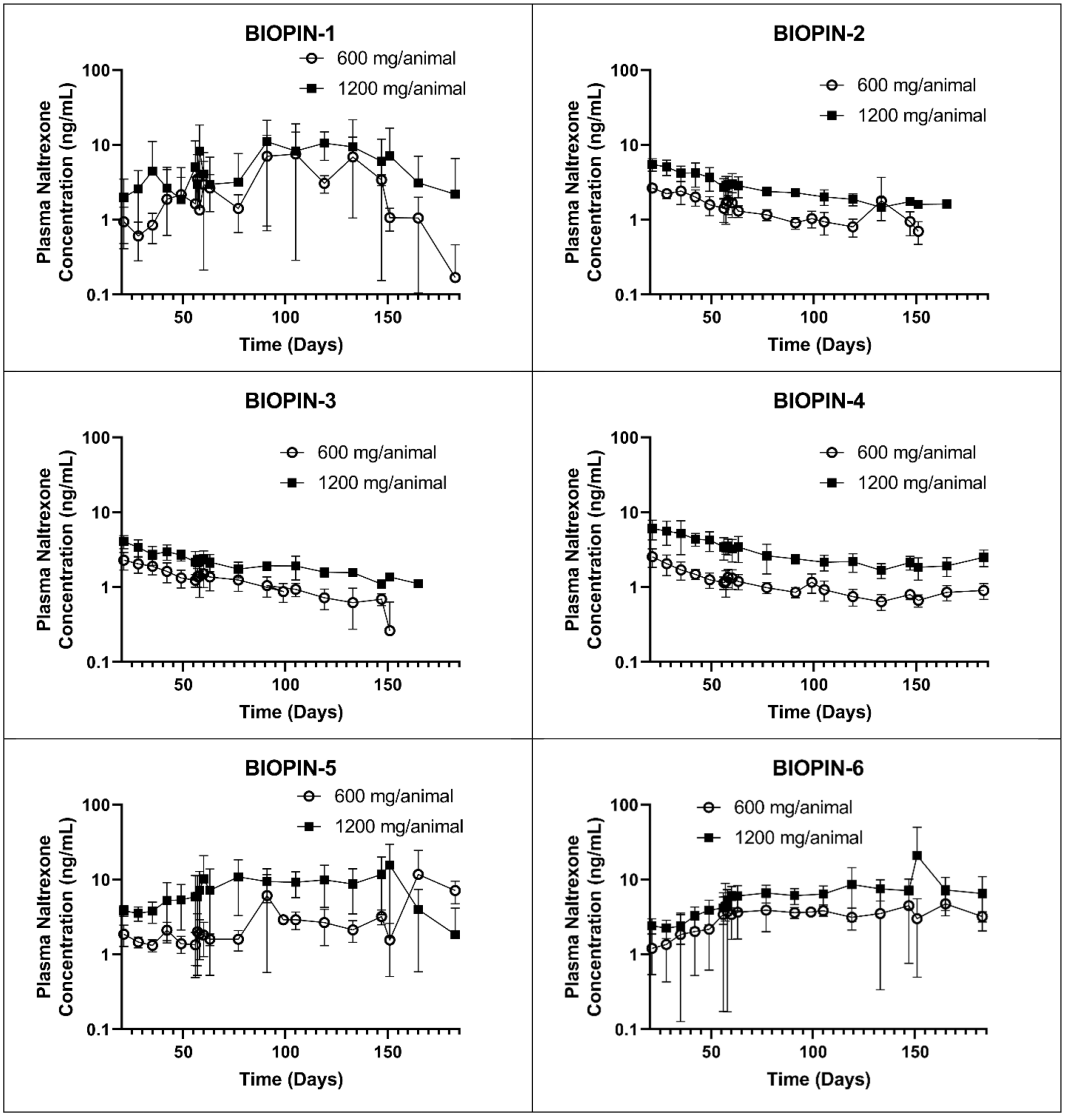
Extended pharmacokinetic evaluation plasma concentrations of naltrexone. BIOPIN implants were evaluated on days 21 through 183. Low‐dose (644 mg; open circle) and high‐dose (1288 mg; filled square) BIOPIN implants are displayed.

The extended‐release profiles for the BIOPIN 2–6 NTX implants, through 6 months, showed a near linear release that was reflected by the naltrexone plasma concentrations (Figure 3). In contrast, the BIOPIN‐1 implants, with PDLLA as the sole release‐controlling polymer, had an erratic release of NTX (Figure 3). On Day 183 post‐implantation, only one out of three animals with the “low dose” BIOPIN‐1 implant had detectable plasma levels of naltrexone. In contrast, all other animals implanted with BIOPIN 2–6 (low and high dose) and the “high dose” BIOPIN‐1 implants maintained plasma naltrexone concentrations above 1.33 ng/mL throughout the 183‐day period, a concentration considered to be therapeutic (National Library of Medicine, 2019).

The plasma concentration of naltrexone with the “high dose” BIOPIN implants was more than twice as high as that of the “low dose” BIOPIN implants. The maximum concentration (*C*_max_) for the low‐dose (644 mg) BIOPIN NTX implants ranged from a low of 12.1 ng/mL for BIOPIN‐6 to 38.5 ng/mL for BIOPIN‐1 (Table 2). The *C*_max_ for the high‐dose (1288 mg) NTX implants ranged from 37.8 ng/mL for BIOPIN‐6 NTX to 80.7 ng/mL for BIOPIN‐1 NTX. The time to achieve *C*_max_ (*T*_max_) was recorded at 3 h for the BIOPIN‐2, 3, and 4 implants (low and high dose) and the BIOPIN‐5 high dose also had a *T*_max_ of 3 h. The BIOPIN‐1 NTX implants reached their *T*_max_ at 632 and 235 h, respectively. While the BIOPIN‐6 NTX implants reached their *T*_max_ at 1230 and 1450 h, respectively. The longest *T*_max_ was 2092 h for the low‐dose BIOPIN‐5 (Table 2).

**FIGURE 4:**
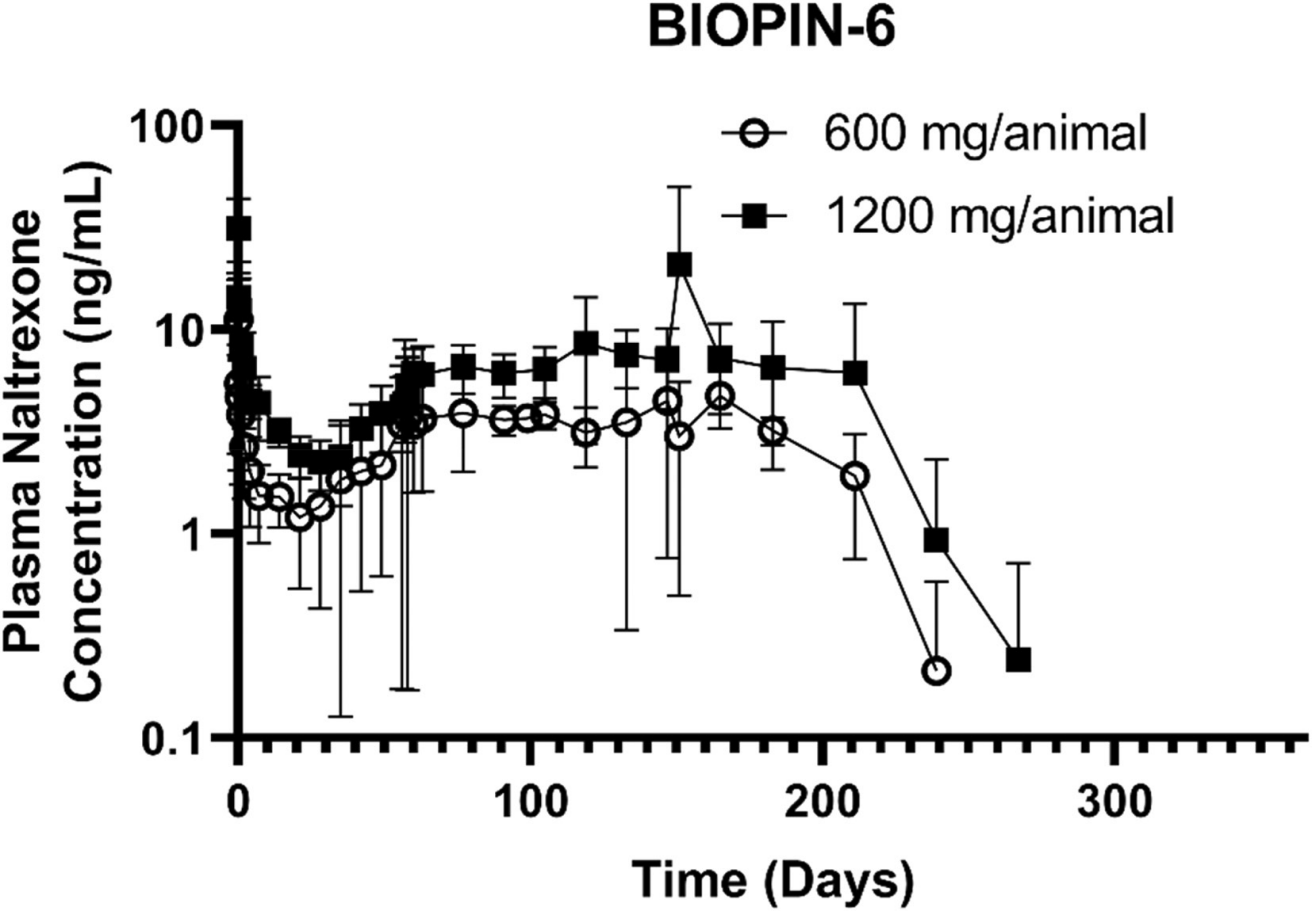
Complete BIOPIN 6 implant pharmacokinetic kinetics profile. Plasma concentrations of naltrexone released are displayed for the low‐dose (644 mg; open circle) and high‐dose (1288 mg; filled square) BIOPIN‐6 implants from Days 0 through 365. Naltrexone plasma levels were below the level of detection on day 267 for the low‐dose BIOPIN‐6 implant and on day 295 post‐implantation for the high‐dose BIOPIN‐6 implant.

Based on the outstanding PK profile of the BIOPIN‐6 NTX implanted animals (Table 2 and Figure 3), the researchers selected the BIOPIN‐6 NTX implanted animals for extended monitoring of plasma naltrexone concentrations through 365 days post‐implantation (Figure 4). The mean plasma concentrations of naltrexone were dose‐dependent and declined over time falling below the limits of quantification by Day 295 (9.8 months). Except for a single mean NTX concentration of 1.21 ng/mL on Day 21 in the low‐dose group, both the low‐ and high‐dose BIOPIN‐6 NTX implants released therapeutic plasma concentrations of naltrexone (>1.33 ng/mL) (National Library of Medicine, 2019) for up to 211 days (6.8 months). Subsequently, the plasma naltrexone concentrations fell below 1.33 ng/mL at 239 days (7.7 months) and became undetectable at 267 days (8.6 months) post‐implantation, remaining undetectable through 365 days (Figure 4). Due to funding constraints on this early‐stage preclinical study, no naltrexone concentration data was obtained for the BIOPIN‐2 and BIOPIN‐3 implants beyond 170 days post‐implantation (Figure 3).

### 3.2 The fentanyl challenges

Thirty‐three canines were selected to undergo the fentanyl challenge, which was performed 97–100 days after implantation. On the day of the challenge, the mean plasma concentrations of naltrexone ranged from 1.2 ng/mL to 9.49 ng/mL (Table 4). Across all NTX BIOPIN NTX implants, the mean NTX concentration was 3.36 ± 1.87 ng/mL (Table 4).

For the fentanyl challenge, it was first necessary to determine the *minimum inhibitory dose of fentanyl* that would cause a reduction in the baseline respiratory rate by >50% from the pre‐dose baseline. For this purpose, a test dose of 30 μg/kg of fentanyl was IV injected into two implant‐naive canines, which are designated as “no implant” in Table 3. This dose was effective, as the respiratory rate for both “no implant” canines rapidly decreased by ~50% but did not result in apnea from the pre‐dose level mean (Table 3). Thus, 30 μg/kg of fentanyl was established as the *minimal inhibitory dose of fentanyl* that would cause a reduction in respiratory rate of ~50%.

**TABLE 3:** Placebo animals challenged with 30 μg/kg of fentanyl.

Fentanyl dose	Time after implantation (days)	BIOPIN number	Dose NTX (mg)	NTX Conc (mg/mL)	Respiration rate (breaths per minute)		
					Predose	30	90
						(s)	(s)
1× (30 μg/kg)	90	1	0	BLQ	40	24	20
	90	2	0	BLQ	32	12	Panting
	90	3	0	BLQ	36	Panting	12
	90	4	0	BLQ	36	Panting	10
	–	NA	0	–	32	28	24
	–	NA	0	–	28	16	12
Mean RR (SD)					34 (4.2)	20 (7.3)	15.6 (6.1)
*p* Value						0.007	0.001

Abbreviations: BLQ, Below the limit of quantitation; min, minutes; mean RR, mean respiration rate; NA, not applicable; SD, Standard deviation; s, seconds.

Four additional animals with placebo BIOPIN implants were then administered the 30 μg/kg dose of IV fentanyl (Table 3). The two “no implant” animals and the four placebo BIOPIN implant animals were combined to create the placebo group (*n* = 6; Table 3).

The fentanyl challenge with a dose of 30 μg/kg of IV fentanyl produced a significant reduction in the respiratory rate at 30 s (*p* = 0.007) and 90 s (*p* = 0.001) in the placebo group (implant naïve [2 canines] + implants without naltrexone [4 canines], *n* = 6 canines total).

**FIGURE 5:**
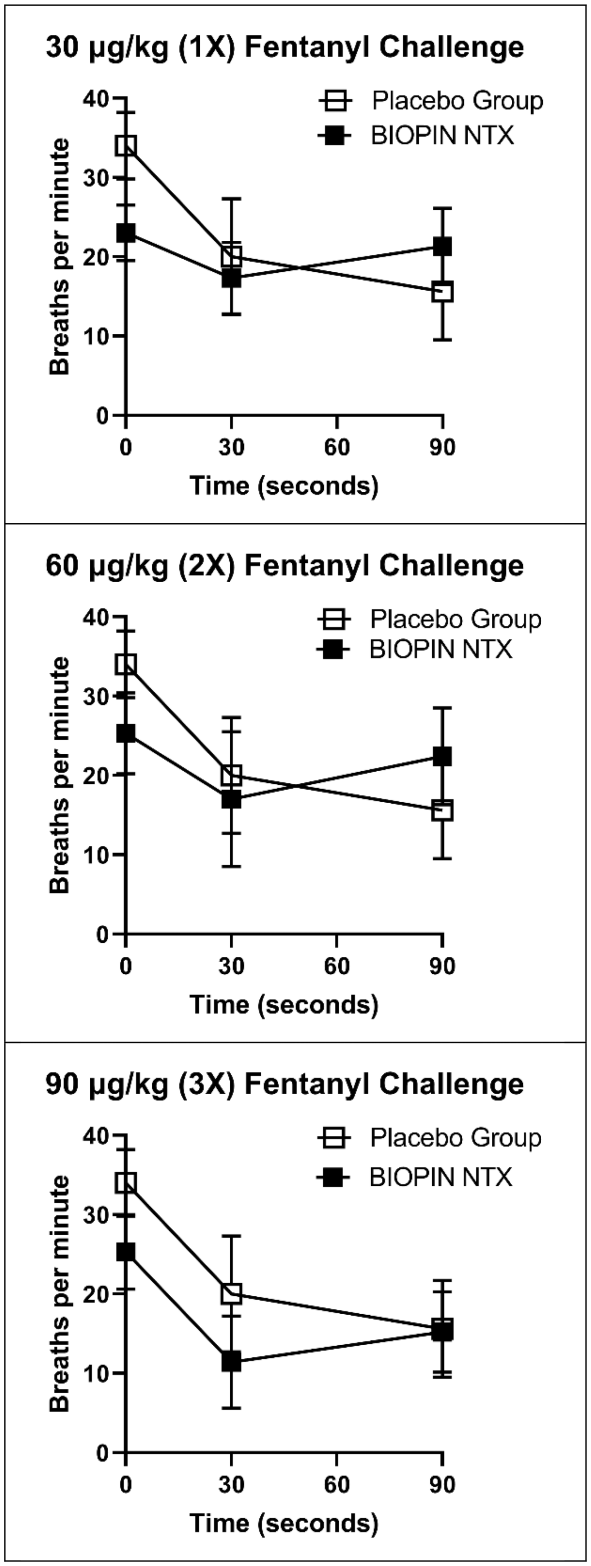
Respiration rates following IV fentanyl challenge for animals with *placebo* group vs. *NTX* BIOPIN implants. The placebo group received 30 μg/kg dose, while the NTX implant group received four escalating doses: 30, 60, 90, and 120 μg/kg of fentanyl. Data are displayed for first 90 s after the IV fentanyl injection.

Next, the canines with BIOPIN NTX implants were challenged with escalating doses of intravenous fentanyl (Table 4). The initial dose was 30 μg/kg, to match the placebo group, then was increased to 60 μg/kg, then to 90 μg/kg and finally to 120 μg/kg (Table 4 and Figure 5).

**TABLE 4:** NTX BIOPIN implants challenged with 30–120 μg/kg of fentanyl.

Fentanyl challenge post implantation							
Fentanyl dose	Time after implantation (days)	BIOPIN number	High or low dose	NTX Conc. (ng/mL)	Respiration rate (breaths per minute)		
					Predose	30 s after fentanyl	90 s after fentanyl
1× (30 μg/kg)	89	2	Low	1.64	20	12	Panting
	90	2	High	2.65	26	20	20
	90	2	High	2.7	24	20	28
	100	3	Low	1.2	20	12	12
	100	3	Low	1.91	20	20	24
	90	4	High	2.6	28	20	16
	89	6	Low	1.94	Panting	24	24
Mean RR (SD)					23 (3.5)	17.3 (4.5)	21.3 (4.8)
*p* Value						0.932	1.0
2× (60 μg/kg)	89	1	High	3.42	32	24	24
	90	1	High	3.16	Panting	Panting	–
	100	2	High	2.14	20	8	16
	89	2	High	4.04	32	8	Panting
	89	3	High	3.3	Panting	28	28
	100	4	High	1.44	20	12	Panting
	100	4	High	1.79	24	16	Panting
	90	5	High	3.17	28	12	16
	90	6	High	4.22	28	–	28
Mean RR (SD)					26.3 (5.1)	17.0 (8.5)	22.4 (6.1)
*p* Value						0.081	0.988
3× (90 μg/kg)	90	1	High	9.49	20	12	8
	100	2	High	2.28	32	22	–
	89	4	High	5.46	32	12	20
	100	5	High	2.91	Panting	Panting	20
	100	5	High	2.12	24	4	Panting
	89	5	High	4.54	28	8	Panting
	90	5	High	6.25	24	16	12
	100	6	High	2.9	24	–	16
	89	6	High	4.2	20	20	20
	90	6	High	5.78	32	16	Panting
Mean RR (SD)					25.3 (4.7)	11.4 (5.8)	15.2 (5.1)
						0.000	0.041
4× (120 μg/kg)	100	6	High	6.13	24	Panting	Panting

Abbreviations: mean RR, mean respiration rate; SD, Standard deviation.

All BIOPIN NTX implanted animals tolerated the IV fentanyl better than the placebo canines at fentanyl doses that ranged from 30 μg/kg up to 90 μg/kg (Table 4). After administering the 30 μg/kg dose, a brief, non‐statistically significant decrease in respiratory rate was observed at 30 90 s (*p* = 0.932 and *p* = 1.0, respectively). The 60 μg/kg group was similar in that there was a slight, nonsignificant decrease in the average respiratory rate (mean RR) that occurred at 30 s (*p* = 0.081) and 90 s (*p* = 0.988) after administering the fentanyl injection. With the 90 μg/kg dosage, there was a severe reduction in the mean RR at 30 s (*p* = 0.000), from the pre‐dose RR of 25.3 (4.7) bpm to 11.4 (5.8) bpm (−55%). However, unlike the placebo group, the BIOPIN NTX implanted canines quickly recovered to 15.2 (5.1) bpm (−40%) at 90 s (Table 4 and Figure 5). No naloxone intervention was required.

Only one animal was challenged with 120 μg/kg of IV fentanyl. It experienced severe respiratory depression that began immediately after the IV fentanyl injection. The canine started panting at 30 and continued to 90 s, followed by a further drop in the respiratory rate to 12 bpm at 3 min and down to 4 bpm at 5 min. This animal required veterinary intervention with two doses of naloxone and oxygen. The staff veterinarian advised against performing additional fentanyl challenges at the 4× dose. Therefore, 90 μg/kg was determined to be the maximum fentanyl dose that was generally safe for the BIOPIN NTX implanted canines in this study.

Panting was observed sporadically in some animals during the fentanyl challenge, both before the initial dose and following the IV fentanyl administration (as shown in Tables 3 and 4). Any animal exhibiting a respiration rate exceeding 40 breaths per minute while at rest, at any point during the fentanyl challenge, was recorded as “panting.” Panting was detected both before the initial dose and at various intervals during the fentanyl challenge. In the placebo group, panting was noted in 19 out of 49 (38.8%) respiratory rate measurements. In the BIOPIN NTX implanted group that received 30 μg/kg of fentanyl, panting was observed in 14 out of the 42 (33.3%) respiratory rate measurements. The 60 μg/kg dosage group saw panting occur in 19 out of 49 (38.8%) measurements, while in the 90 μg/kg dosage group, panting was recorded in 19 out of 62 (30.6%) measurements.

## 4 DISCUSSION

Extended‐release BIOPIN naltrexone (NTX) implants showed protective benefits against severe respiratory depression caused by intravenous fentanyl in canines, in contrast to the placebo cohort. This protective effect was present at approximately 3.5 months after the subcutaneous implantation, even when the dose of fentanyl administered was three times higher than the placebo group. These results matched those from previous experiments on rodents conducted by our group. In that study, the NTX implants provided a similar protective effect against fentanyl‐induced respiratory depression (FIRD) (Benner et al., 2023).

**FIGURE 6:**
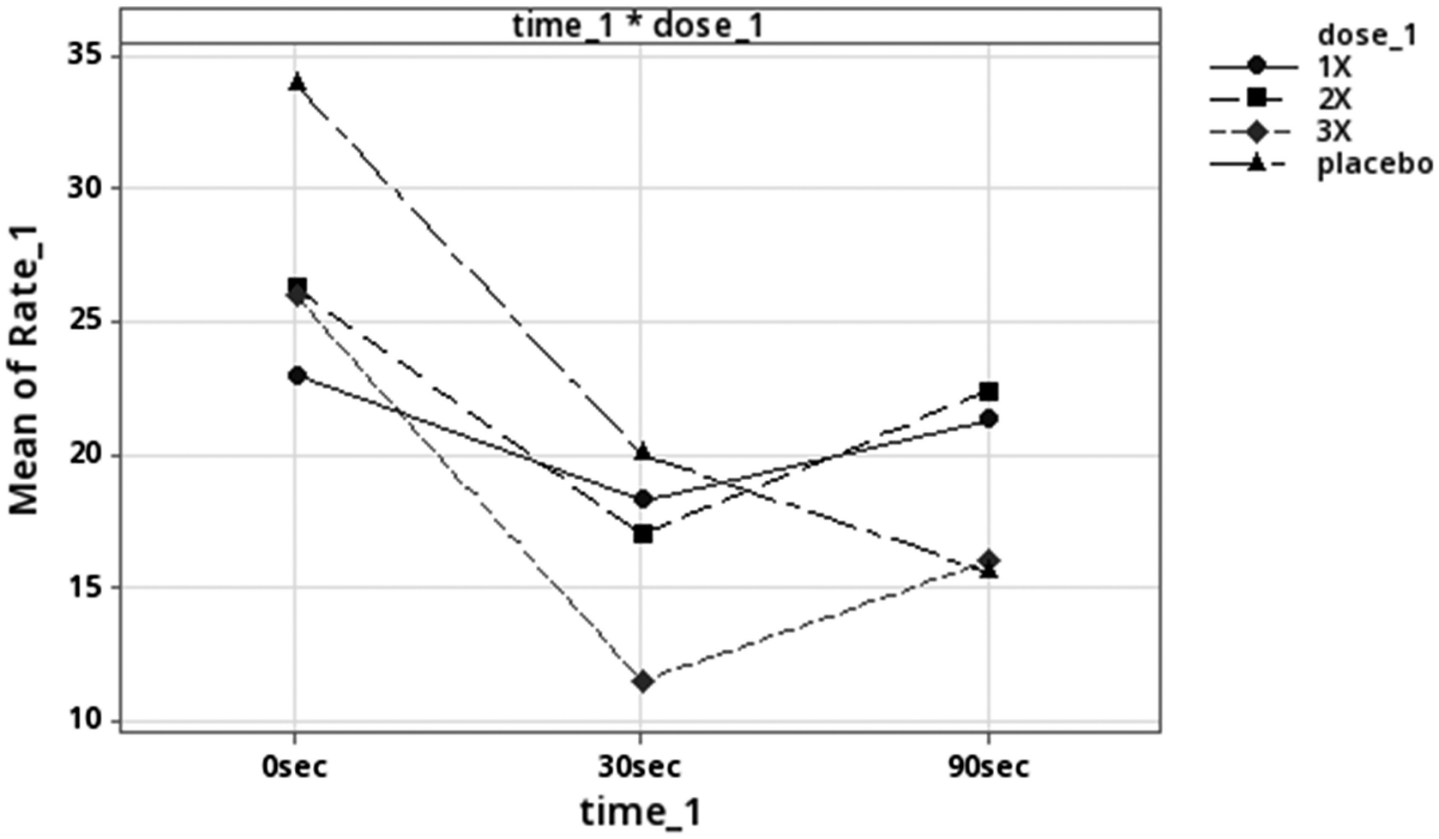
Respiration rates following IV fentanyl challenge for animals with *placebo* group versus *NTX* BIOPIN implants. The placebo group received 30 μg/kg dose, while the NTX implant group received three escalating doses of fentanyl: 30, 60, 90 μg/kg. Data are displayed for the first 90 s after the IV fentanyl injection. Data suggests BIOPIN implants mitigated the respiratory depression seen at 90 s for the 1× and 2× doses, but not the 3× dose. The 0 s represents the pre‐dose time.

**FIGURE 7:**
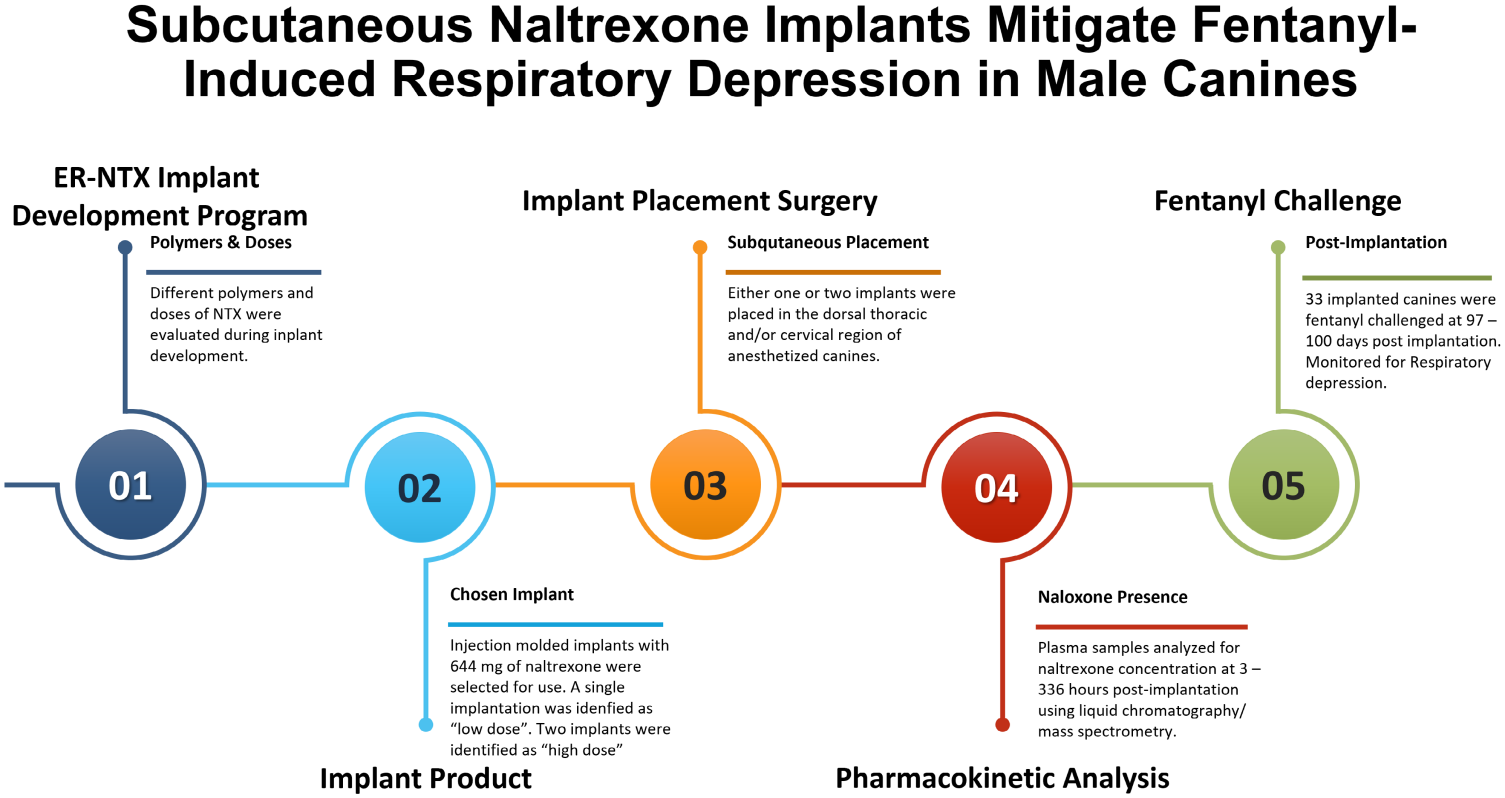
A schematic timeline illustrating the sequence of experimental steps performed throughout this project.

The placebo group (composed of implant‐naïve and placebo implanted animals) experienced a severe and rapid (~ 50%) reduction in respiratory rate (RR) after the 30 μg/kg dose of fentanyl, which was determined to be the *minimum inhibitory dose of fentanyl*. In contrast, canines with BIOPIN NTX implants mitigated the respiratory depression induced by the fentanyl challenge. When the NTX‐implanted canines received the 60 μg/kg dose of intravenous fentanyl, there was a brief, nonsignificant decrease in their average respiratory rate (less than 50%) at 30 s, from which they recovered completely by 90 s. With the 90 μg/kg dose of fentanyl, there was a momentary and severe drop in the average respiratory rate at 30 s, but again, the canines with BIOPIN NTX implants rapidly recovered by 3 min (Figure 5). The protective effect of the BIOPIN NTX implants can be clearly seen in Figure 6 at 90 s for the 30 μg/kg and 60 μg/kg doses, but not the 120 μg/kg dose (Figure 7).

These results are consistent with results of our earlier rodent study. In that study, an intravenous injection of 4 mcg of fentanyl caused a profound reduction in the respiration rate of the placebo‐implanted rats. It dropped from 208 ± 14 breaths/minute at pre‐dose to 84 ± 12 breaths/minute (*p* = 0.0003) at 2 min. In contrast, all naltrexone implanted animals easily tolerated *twice* the dose of 8 mcg of fentanyl without any significant reduction in respiration rate (Benner et al., 2023).

The protective effect of the NTX implants had a dose limit. A single animal was administered a 120 μg/kg dose of fentanyl and experienced a profound reduction in RR that required two doses of IV naloxone to recover, despite having a high‐dose NTX BIOPIN 6 implant. Its plasma concentration on the day of the fentanyl challenge was highly therapeutic at 6.13 ng/mL, yet this concentration was insufficient to block the FIRD from the 120 μg/kg dose of IV fentanyl.

There was a suggestion that the efficacy of the BIOPIN NTX implants was dependent on the plasma concentration of NTX at the time that the IV fentanyl was injected. This pattern was convincing but not definite. Canines with the lowest plasma concentrations (<2 ng/mL) had more severe reductions in their respiratory rates when compared to their fellow canines with higher plasma levels of NTX. For instance, in the 30 μg/kg dosing cohort, the BIOPIN 3‐low‐dose implant dog had the lowest plasma naltrexone concentration of 1.2 ng/mL. That animal experienced the most dramatic reduction in its respiratory rate of its 30 μg/kg fentanyl cohort peers (Table 4). Similarly, another animal with the BIOPIN 2‐low‐dose implant had a low plasma NTX concentration (1.64 ng/mL) and had a profound reduction in respiratory rate after the 30 μg/kg fentanyl dose, followed by panting. In the 60 μg/kg dose cohort, the animal with a BIOPIN 4‐high‐dose implant had a serum concentration of 1.44 ng/mL and showed a notable reduction in its respiratory rate. Canines with serum concentrations higher than >2 ng/mL were protected from FIRD. There is a paucity of animal‐based pharmacokinetic (PK) investigations of extended‐release NTX implants in the literature. We found two rodent studies for comparison but no canine studies. In 1986, Yoburn et al. implanted two 30 mg naltrexone pellets subcutaneously in rats. Their PK results showed an initial “burst” release, with a very high plasma NTX level of 350 ng/mL at 1 h. The NTX concentration declined to 24 ng/mL over the next 192 h. The authors concluded that NTX implants could release naltrexone systemically for at least 192 h (8 days) after implantation (Yoburn et al., 1986). Bartus et al. administered biodegradable poly (lactide co‐glycolic acid) microspheres loaded with 35% naltrexone to rats (*n* = 8), with the rats observed for a month. In this study, the NTX dose was 50 mg/kg (~22.5 mg/rat), and this formulation is now commercially known as Vivitrol™. Their peak NTX plasma levels were like our low‐dose implants, but with a duration of 1 month. Their peak NTX plasma levels measured 15–19 ng/mL on day 3, decreasing to 12 ng/mL by day 8, and becoming undetectable by day 35 (Bartus et al., 2003).

Caution is necessary when extrapolating results from animal studies to humans. However, given the scarcity of animal‐based pharmacokinetic (PK) studies on extended‐release naltrexone (NTX) implants, human data comparisons can be valuable. A 2010 Australian study compared oral naltrexone with sustained‐release implant naltrexone and examined the relationship between plasma naltrexone levels and heroin cravings (Hulse et al., 2010). This study predated the introduction of fentanyl into the illicit drug market. It found that sustained‐release implant naltrexone was more effective than oral naltrexone in preventing heroin relapse among heroin‐dependent individuals. Blood naltrexone concentrations below 0.5 ng/mL were associated with a 2.5 times higher risk of withdrawal symptoms and relapse. Patients with blood naltrexone levels of 1–3 ng/mL experienced lower heroin cravings and a 35% reduced relapse rate. The study concluded that the optimal blood level for naltrexone is 1–3 ng/mL, with levels below 0.5 ng/mL being ineffective (Hulse et al., 2010). Vereby and colleagues demonstrated that plasma concentrations above 2 ng/mL could fully antagonize the effects of 25 mg intravenous heroin (Verebey et al., 1976). Additionally, the primary outcome for the phase 1 clinical trial of the O'Neil Long‐Acting Naltrexone Implant (OLANI), containing 1.8 grams of NTX, was the percentage of participants maintaining NTX blood levels above 1.33 ng/mL for 180 days (ClinicalTrials.gov, 2022; NCT 03810495). These human studies indicate that NTX plasma concentrations above 1 ng/mL are effective at reducing opioid cravings, preventing relapse, and blocking the effects of intravenous heroin. The FDA has accepted a concentration of above 1.33 ng/mL as a PK comparator. None of these studies assessed the impact of intravenous fentanyl on respiratory status in patients with existing extended‐release NTX implants. These human NTX PK reports align with our observation that serum NTX concentrations above 2 ng/mL were protective against fentanyl‐induced respiratory depression (FIRD) in the male canines in our study.

Panting is defined as a respiratory rate of >40 breaths per minute while at rest in canines (Lake Normal at Mooresville Animal Hospital, 2023). In veterinary clinics, up to 78.5% of dogs become stressful or fearful upon entering the clinic, and 29% of dogs manifest extreme stress (Vaisanen et al., 2005). Furthermore, dogs that are caged prior to surgery are more likely to exhibit symptoms of fear, stress, and anxiety that include panting, snout licking, barking, or howling (Edwards et al., 2019). This clarifies why our animals panted before receiving the dose. In total, roughly 1/3 of our dogs showed panting at some point during the observation period, which included prior to receiving the dose and after the IV fentanyl administration. Panting after administration of opioids is well described in the literature. Dogs often pant more after receiving opioids. Opioids stimulate the area of the hypothalamus that is responsible for temperature regulation, leading to increased panting and a drop in body temperature (Bartus et al., 2003).

Interestingly, morphine and fentanyl have similar mu opioid receptor binding affinities. Morphine has a receptor binding affinity of 1.168 nM, while fentanyl has an affinity of 1.346 nM. However, fentanyl is much more lipophilic with a 4.28 log P, as compared to morphine with a 1.07 log P (Moss & Carlo, 2019). The high lipophilicity of fentanyl produces a more rapid onset of action within 2 min as compared to morphine, which has an onset of 6 min. Some have called for making a higher dose of naloxone available for emergency medical technicians (Moss & Carlo, 2019). This study suggests that extended‐release opioid antagonists such as the naltrexone implants used in this study, might someday offer “real world” protection by blocking FIRD after IV fentanyl injection.

This study had several strengths. The XR‐NTX implants used in the study consistently released significant levels of naltrexone for 3.5 months after implantation. Most animals maintained therapeutic plasma levels of naltrexone on the day of the fentanyl challenge, which effectively reduced the respiratory depression seen in the placebo group. Additionally, there was an indication that a minimum plasma concentration of 1.8 ng/mL might be necessary to counteract FIRD. Importantly, this research confirmed our previous findings from a rodent study in a larger mammal more like humans. The results demonstrated that BIOPIN NTX implants with extended‐release capabilities can effectively mitigate fentanyl‐induced respiratory depression in canines for an extended period after implantation.

This study also had limitations. One limitation was that we only measured the respiratory rate and didn't have the capability to measure other aspects of respiratory function like tidal volume. We couldn't use more precise measures like arterial blood gases because it would have required sedating the animals, which we intentionally avoided. We attempted to use pulse oximetry, but this was unsuccessful because the oximeters fell off whenever the conscious dogs moved. Additionally, panting, which caused some inaccuracies in measuring the respiratory rates, occurred in about 1/3 of all the respiratory measurements, including the pre‐dose measurements. Given these challenges, canines may not be the ideal animal model for future fentanyl challenge experiments.

This study in male dogs confirmed the potential of BIOPIN implants to mitigate fentanyl‐induced respiratory depression, about 3 months after implantation. The results were noteworthy. The placebo group experienced rapid and severe respiratory depression after a 30 μg/kg dose of IV fentanyl. In contrast, the naltrexone‐implanted dogs displayed impressive resilience, tolerating doses as high as 90 μg/kg without experiencing significant respiratory depression. Bioabsorbable, extended‐release naltrexone implants have proven effective in reducing fentanyl‐induced respiratory depression in experimental canine subjects. This technology holds promise for mitigating fentanyl‐induced respiratory depression in human subjects.

## FUNDING INFORMATION

This work was supported by the National Institutes of Health, National Institute on Drug Abuse: grant UG3 DA048338.

## CONFLICT OF INTEREST STATEMENT

JDB and SMC have a financial interest in The Drug Delivery Company, LLC, which is the licensee of USPTO# 11,197,819 B1. MF has been a consultant for The Drug Delivery Company LLC. JAH, RLJ, DD, and VH have no relevant financial disclosures.

## ETHICS STATEMENT

Ethics approval statement, patient consent statement, permission to reproduce material from other sources, and clinical trial registration are not applicable.

## Data Availability

The data that support the findings of this study are available from the corresponding author upon reasonable request.
